# Cryptolepine, a Plant Alkaloid, Inhibits the Growth of Non-Melanoma Skin Cancer Cells through Inhibition of Topoisomerase and Induction of DNA Damage

**DOI:** 10.3390/molecules21121758

**Published:** 2016-12-21

**Authors:** Harish C. Pal, Santosh K. Katiyar

**Affiliations:** 1 Department of Dermatology, University of Alabama at Birmingham, Birmingham, AL 35294, USA; 2 Environmental Health Sciences, University of Alabama at Birmingham, Birmingham, AL 35294, USA; 3 Comprehensive Cancer Center, University of Alabama at Birmingham, Birmingham, AL 35294, USA; 4 Birmingham Veterans Affairs Medical Center, Birmingham, AL 35294, USA

**Keywords:** cryptolepine, skin cancer, topoisomerase, DNA damage, cell cycle, apoptosis

## Abstract

Topoisomerases have been shown to have roles in cancer progression. Here, we have examined the effect of cryptolepine, a plant alkaloid, on the growth of human non-melanoma skin cancer cells (NMSCC) and underlying mechanism of action. For this purpose SCC-13 and A431 cell lines were used as an in vitro model. Our study reveals that SCC-13 and A431 cells express higher levels as well as activity of topoisomerase (Topo I and Topo II) compared with normal human epidermal keratinocytes. Treatment of NMSCC with cryptolepine (2.5, 5.0 and 7.5 µM) for 24 h resulted in marked decrease in topoisomerase activity, which was associated with substantial DNA damage as detected by the comet assay. Cryptolepine induced DNA damage resulted in: (i) an increase in the phosphorylation of ATM/ATR, BRCA1, Chk1/Chk2 and γH2AX; (ii) activation of p53 signaling cascade, including enhanced protein expressions of p16 and p21; (iii) downregulation of cyclin-dependent kinases, cyclin D1, cyclin A, cyclin E and proteins involved in cell division (e.g., Cdc25a and Cdc25b) leading to cell cycle arrest at S-phase; and (iv) mitochondrial membrane potential was disrupted and cytochrome c released. These changes in NMSCC by cryptolepine resulted in significant reduction in cell viability, colony formation and increase in apoptotic cell death.

## 1. Introduction

**Figure 1:**
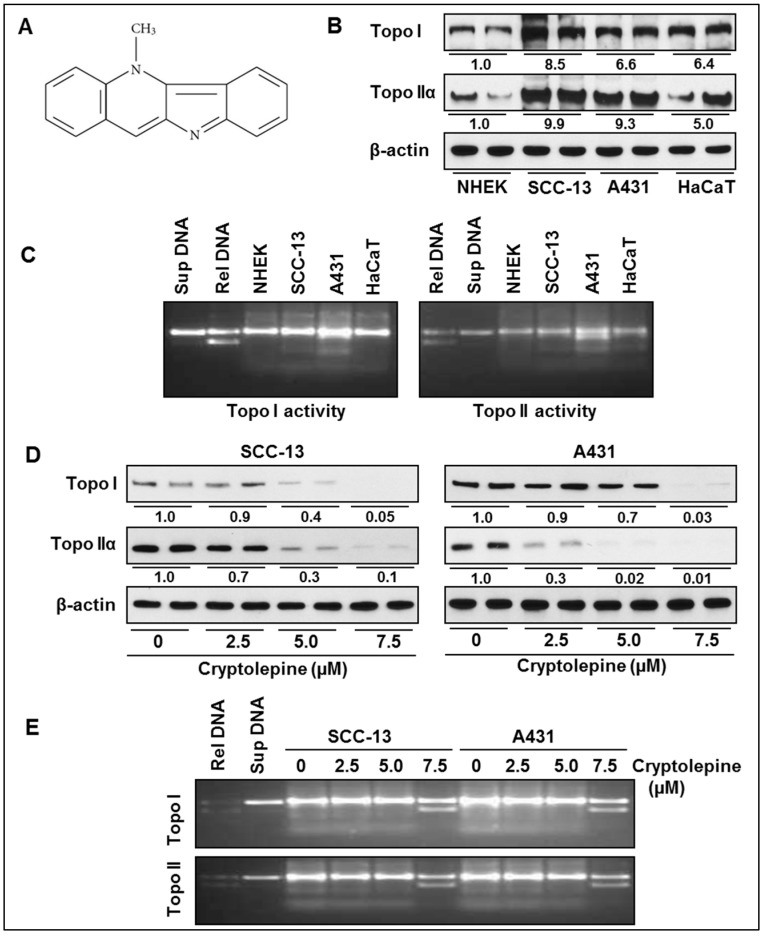
Comparison of basal expression and activity of topoisomerases in non-melanoma skin cancer (NMSC) cell lines, and effect of cryptolepine on topoisomerase in NMSC cells. (**A**) Molecular structure of cryptolepine, a plant alkaloid; (**B**) Basal expression of topoisomerases (Topo I and Topo IIα) in various cell lines was determined in total cell lysates using western blot analysis; (**C**) Topoisomerases containing cell extracts were subjected to the analysis of enzyme activity using topoisomerase activity assay kit, as detailed in Materials and Methods; (**D**) SCC-13 and A431 cells were treated with various concentrations of cryptolepine (0, 2.5, 5.0, and 7.5 μM) for 24 h, total cell lysates were subjected to western blot analysis for the detection of Topo I and Topo IIα. The numerical value of band density is shown under blot, and the band density of control was arbitrarily selected as 1 and comparison was then made with densitometry values of other treatment groups; (**E**) Cell extracts containing topoisomerases from different treatment groups were subjected to the analysis of enzyme activity using topoisomerase activity assay kit. Topo = topoisomerase, Sup DNA = Supercoiled DNA, Rel DNA = Relax DNA.

Cryptolepine (Figure 1A) is an alkaloid isolated from the roots of Central and West African shrub *Cryptolepis sanguinolenta* (Lindl.). The aqueous extract from the roots of this plants have been traditionally used for the treatment of malaria, rheumatism, urinary tract infections, upper respiratory tract infections and intestinal disorders in Central and West African countries like Ghana and Nigeria [1,2]. Cryptolepine has also demonstrated various pharmacological and biological activities including anti-malarial [3], anti-bacterial [4], anti-fungal [5], and anti-hyperglycaemic [6,7] activities. The anti-inflammatory activity of cryptolepine has been documented in different animal model systems [8,9]. The anti-inflammatory activity of cryptolepine is due to inhibition of COX-2/PGE_2_ signaling and inhibition of other promotors of inflammation including TNFα and iNOS [8,9,10,11]. Since chronic and persistent inflammation is closely associated with development and progression of variety of cancers, attempts have been made to evaluate antitumor potential of cryptolepine. Studies have demonstrated that cryptolepine possesses cytotoxic potential against mammalian cancer cells [12,13,14]. However, the molecular mechanisms of potential toxicity against cancer cells are not fully understood. Some studies have suggested that the mechanism by which cryptolepine exhibits anticancer potential may be through its direct binding to DNA and inhibition of DNA synthesis or inhibition of topoisomerase II (Topo II) [15,16,17]. 

Topoisomerases are highly specialized nuclear enzymes involved in the removal of superhelical tension on chromosomal DNA, correction of topological DNA errors during replication, transcription, recombination and chromosomal condensation [18,19]. Topoisomerases act by sequential breakage and reunion of either one stand of DNA or both the strands of DNA depending upon the type of topoisomerase involved in the process [20,21]. Moreover, in the absence of topoisomerase functions, positive supercoiling of DNA rapidly stalls the replication and transcription, and negative supercoiling generates abnormal DNA structures [22,23]. These topological changes in DNA may result in activation or repression of gene transcription. In fact inhibition of topoisomerase action particularly topoisomerase II inhibition is the central mechanism of various anticancer agents. Inhibition of topoisomerase II may lead to alteration in DNA structure and DNA damage and ultimately the induction of apoptotic cell death [21,22]. Non-melanoma skin cancers (NMSC) are the most commonly diagnosed cancers in the United States [24,25]. It is estimated that >2.0 million Americans are diagnosed each year with NMSC, and about 2000 people are estimated to die from this malignancy every year. The chronic exposure to solar ultraviolet (UV) radiation is considered as a major etiological factor for this disease. Due to change in life style, incidence of NMSCs is rising continuously due to immunosuppressive, inflammatory and oxidative stress caused by UV radiation exposure. Moreover, patients with organ transplants are at ~100-fold greater risk for the development of skin cancer as compared to healthy individuals. Because of increasing risk of NMSC, more potent, safe and affordable anticancer strategies are required for its prevention and/or treatment. In the present study, therefore, we are assessing the anti-skin cancer effect of cryptolepine using two major and commonly used NMSC cell lines SCC-13 and A431 as an in vitro model.

## 2. Results

### 2.1. Basal Expression and Activity of Topoisomerases in NMSC Cells 

First we determined and compared the basal levels and activities of topoisomerases (I and II) in NMSCs cells (SCC-13 and A431) and data were compared with the NHEK and immortalized HaCaT cells. Western blot analysis revealed that basal levels of topoisomerases (Topo I and Topo IIα) were higher in SCC-13 and A431 cells compared to NHEK (Figure 1B). Interestingly, the expression levels of Topo I and Topo IIα were also higher in HaCaT cells comparted to NHEK and the levels were approximately similar to that of NMSC cells (Figure 1B). Moreover, the gel electrophoresis data indicated that the Topo I and Topo II activity was greater in SCC-13 and A431 cells compared to NHEK and HaCaT cells (Figure 1C). Band density reflects the activity of the enzyme.

### 2.2. Cryptolepine Inhibits Topoisomerase Expression and Activity in NMSC Cells

It has been suggested that higher expression and activity of topoisomerases in cancer cells may facilitate enhanced and uncontrolled proliferative potential and survival of these cells [19,20,23], therefore, we determined the effect of cryptolepine on topoisomerase expression and activities in SCC-13 and A431 cells. Western blot analysis revealed that the treatment of NMSC cells with cryptolepine reduced the levels of Topo I and Topo IIα in both cell lines (Figure 1D) compared to non-cryptolepine treated control cells. Treatment of cryptolepine also inhibited the activities of topoisomerases in SCC-13 and A431 cells, as reflected from the gel electrophoresis data (Figure 1E). The inhibitory effect of cryptolepine was greater on Topo IIα than Topo I in NMSC cells.

### 2.3. Cryptolepine Induces DNA Damage in NMSC Cells

**Figure 2:**
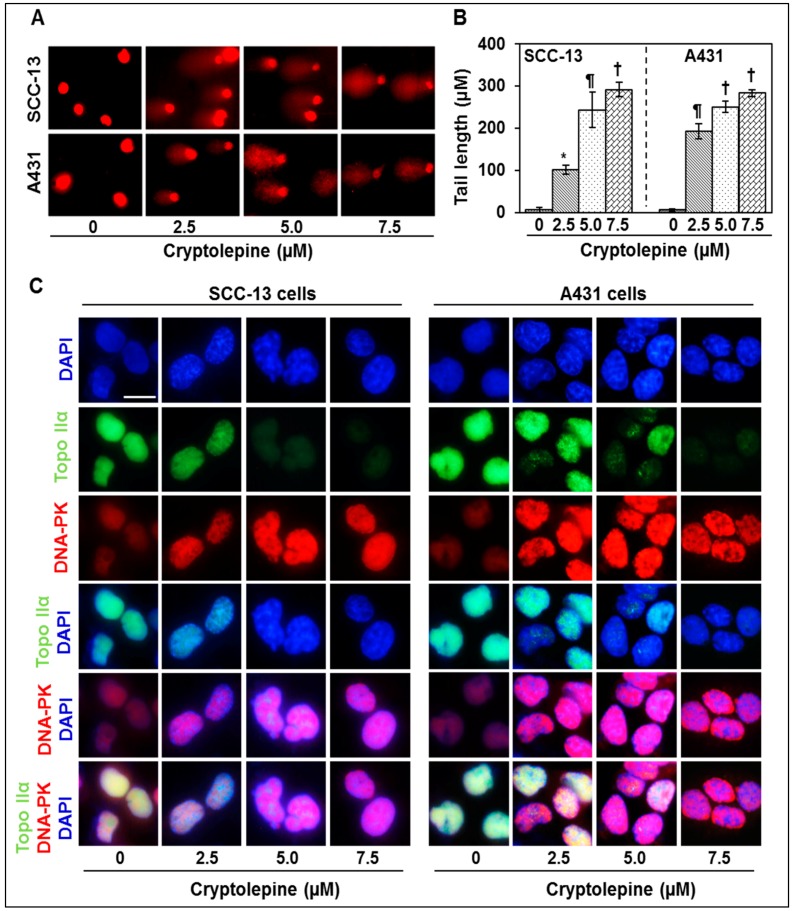
Treatment of NMSC cells with cryptolepine induces DNA damage which is associated with inhibition of topoisomerase IIα expression and induction of DNA-PK protein expression. (**A**) Cryptolepine (0, 2.5, 5.0 and 7.5 µM for 24 h) induced DNA damage was determined by comet assay, as described in Materials and Methods; (**B**) Comet’s tail length was measured using opencomet software. Data are expressed in terms of tail length as mean ± SD, *n* = 6. Statistical significance versus control, * *p* < 0.05; ^¶^ *p* < 0.01; ^†^ *p* < 0.001; (**C**) Approximately 5 × 10^4^ SCC-13 or A431 cells/well of four chambered slide were treated with 0, 2.5, 5.0 and 7.5 µM of cryptolepine for 24 h and stained with topoisomerase IIα- and DNA-PK-specific primary antibodies for double staining, as detailed in Materials and methods. After washing with PBS, slides were mounted with vectashield mounting media containing DAPI. Representative photomicrographs are shown. Bar size = 5 µm.

Topo IIα in particular catalyzes the interconversion of topological isomers of DNA through a transient double strand DNA break, and is followed by double-strand passing and religation. Therefore inhibition of Topo IIα function will result in severe DNA damage. Moreover, induction of DNA damage through inhibition of topoisomerase activity is the major mechanism of anticancer drugs [19,20,23]. As cryptolepine inhibits Topo I and Topo II activity, we determined its effect on DNA damage in SCC-13 and A431 cells using Comet assay. Comet assay analysis indicated that treatment of SCC-13 and A431 cells with cryptolepine induces significant DNA damage (*p* < 0.05 to *p* < 0.001) which is reflected from the comet tail length in cryptolepine- treated cells compared to non-treated control cells and this effect was dose-dependent (Figure 2A,B).

The DNA-dependent protein kinase (DNA-PK), a nuclear serine/threonine protein kinase and crucial component of the DNA double-strand break repair machinery, is known to be activated upon association with DNA in response to DNA damage [26,27]. Therefore, expression of DNA-PK in cryptolepine treated cells was analyzed using immunohistochemistry. Results of Topo IIα and DNA-PK double staining clearly demonstrated that DNA-PK expression was greatly enhanced whereas the expression of Topo IIα was reduced in cryptolepine treated SCC-13 and A431 cells (Figure 2C).

### 2.4. Cryptolepine Enhances the Expression of DNA Damage Response Mediator and Effector Cascade in NMSC Cell

**Figure 3:**
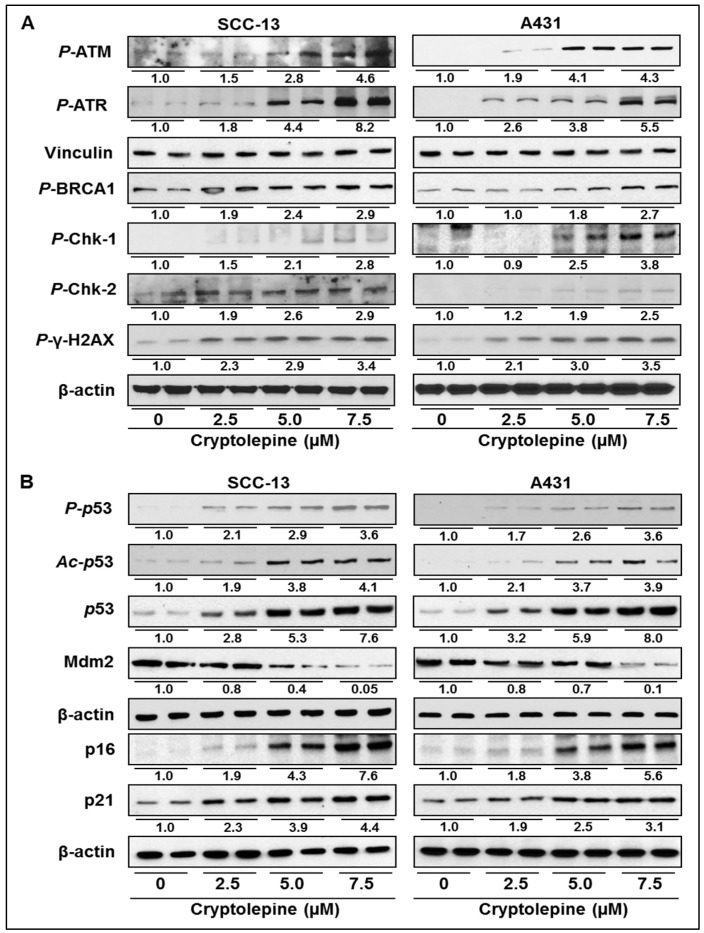
Treatment of cryptolepine enhances the expressions of DNA damage response mediators and effector cascade in NMSC cells. (**A**) Cryptolepine treatment enhances expressions of DNA damage response proteins in SCC-13 and A431 cells; and (**B**) DNA damage effectors in SCC-13 and A431 cells. Cells were treated with cryptolepine (0, 2.5, 5.0 and 7.5 µM) for 24 h. Cell lysates were subjected to western blot analysis for determination of the levels of different protein biomarkers. Blots were developed with chemiluminescence-specific ECL. The numerical value of band density is shown under blot, and the band density of control was arbitrarily selected as 1 and comparison was then made with densitometry values of other treatment groups, as detailed under Materials and Methods. Equal loading of proteins was confirmed by stripping the membranes and probed with β-actin or vinculin.

DNA damage response is initiated by variety of protein kinases and accessory factors such as ATR, ATM, and DNA-PK. These kinases/factors have ability to sense the DNA damage. In the event of DNA damage, these molecules respond by phosphorylating and activating downstream factors such as Chk1, Chk2, p53 and associated protein factors involved in the process of DNA repair, cell cycle progression and apoptosis. Therefore, we determined the effect of cryptolepine on phosphorylation of these DNA damage response factors. We found that cryptolepine treatment induced phosphorylation of ATM, ATR, BRCA1, γH2AX, Chk1 and Chk2 in SCC-13 and A431 cells in a dose-dependent manner (Figure 3A). Representative data are produced from two separate experiments. Cellular responses to DNA damage are known to be mediated by affecting the cell cycle progression, inducing cellular senescence and apoptosis to eliminate the damaged cells.

The tumor suppressive protein p53 plays a crucial role in DNA damage response, cell cycle progression and apoptosis [28,29]. Expression and activation of p53 is negatively regulated by mdm2 protein. We have found that cryptolepine induced DNA damage in SCC-13 and A431 cells resulted in concentration-dependent increase in p53 phosphorylation and acetylation (Figure 3B). The activation of p53 was observed due to downregulation of mdm2 expression leading to accumulation of p53 protein in cells (Figure 3B). Further, western blot analysis revealed that treatment of cryptolepine enhanced the expressions of tumor suppressor p16 and p21 proteins in SCC-13 and A431 cells (Figure 3B).

### 2.5. Cryptolepine Induces S-Phase Cell Cycle Arrest in NMSC Cells

As we found a significant DNA damage in NMSC cells after a treatment with cryptolepine, we determined the possible inhibitory effect of cryptolepine on cell cycle progression in SCC-13 and A431 cells.

**Figure 4:**
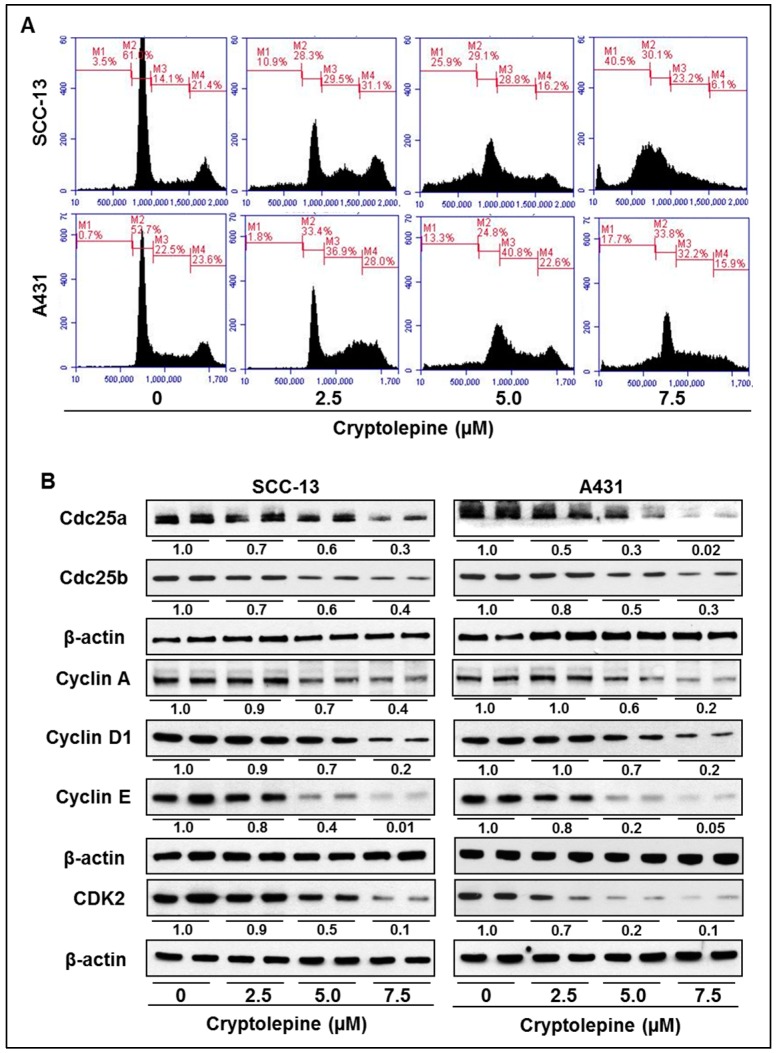
Treatment of cryptolepine induces S-phase cell cycle arrest in NMSC cells. (**A**) Approximately 2 × 10^5^ SCC-13 or A431 cells were treated with different doses of cryptolepine (0, 2.5, 5.0 and 7.5 µM) for 24 h. After harvesting the cells, cells were stained with propidium iodide and analyzed on Accuri Q6 flow cytometer for DNA content in different phases of cell cycle. M3 compartment shows the cells in S-phase; (**B**) Cell lysates from cryptolepine treated and non-treated controls of SCC-13 and A431 cells were subjected to western blot analysis to determine the effect on expression of cell cycle regulatory proteins. The numerical value of band density is shown under blot, and the band density of control (non-treated group) was arbitrarily selected as 1 and comparison was then made with densitometry values of other treatment groups.

Representative data are produced from two separate experiments. As summarized in Figure 4A, treatment of SCC-13 cells with cryptolepine for 24 h resulted in accumulation of cells in S-phase (M3 compartment) at the concentrations used, 2.5 µM (29.5%), 5.0 µM (28.8%), and 7.5 µM (23.2%) compared with non-cryptolepine-treated control cells (14.1%). Importantly, the accumulation of cells in S-phase is reducing though insignificantly with the increase of the concentration of cryptolepine. It may be because of induction of cryptolepine-induced apoptosis in G0 phase (M1 compartment) of cell cycle at the same time as is evident by the histograms (Figure 4A). Similar effects of cryptolepine on S-phase arrest were also found in A431 cells (Figure 4A). These data suggest that induction of DNA damage in SCC-13 and A431 cells by cryptolepine is associated with the increases in apoptotic cell death (G0 phase) and accumulation of cells in S-phase that resulted in dysregulation of cell cycle progression. Progression of cell cycle is a highly regulated process. It involves variety of regulatory check-points, such as cyclins, cell division cycle (Cdc25), cyclin-dependent-kinases (CDKs) and inhibitor of CDKs (e.g., p16/p21) [30,31]. In the present study, we found that as a consequence of cryptolepine induced DNA damage response signaling and cell cycle arrest, expression levels of Cdc25a and Cdc25b were also decreased in SCC-13 and A431 cells (Figure 4B). It was also found that cryptolepine induced S-phase arrest was accompanied by downregulation of cyclin A, cyclin D1, cyclin E and CDK2 protein expressions (Figure 4B). It has been demonstrated that in the event of DNA damage, activated p16 and p21 binds to CDK/cyclin complexes to inhibit cell cycle progression. These observations suggest that the cryptolepine-induced enhancement of the levels of CDK inhibitors (p16 and p21, Figure 3B) plays an important role in the cryptolepine-induced S-phase arrest of cell cycle progression in NMSC cells.

### 2.6. Cryptolepine Induces Disruption of Mitochondrial Membrane Potential in NMSC Cells

**Figure 5:**
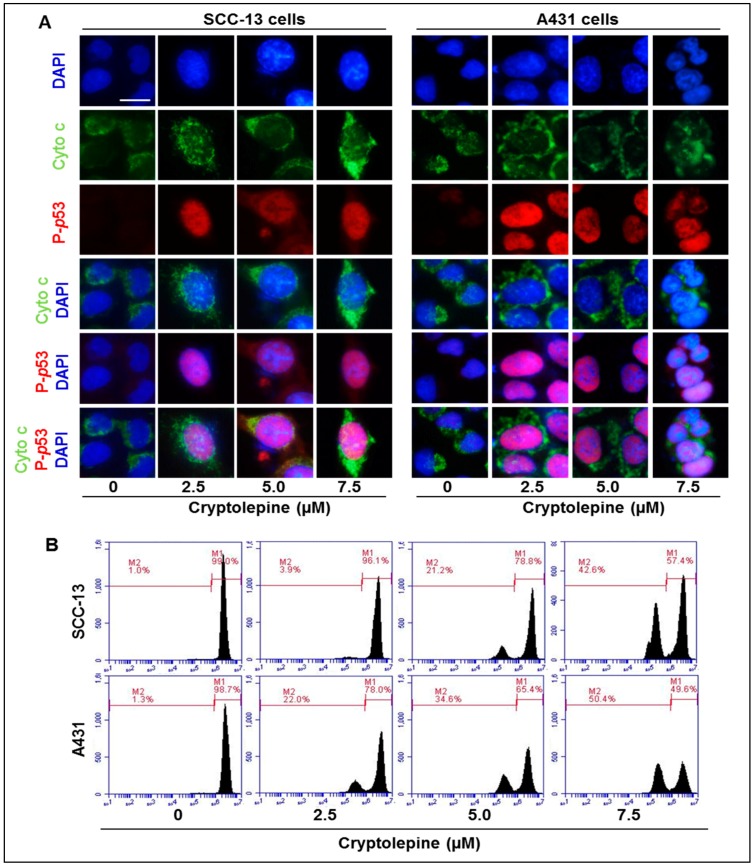
Cryptolepine treatment stimulates the loss of mitochondrial membrane potential and subsequently release cytochrome c in NMSC cells. (**A**) SCC-13 or A431 cells were treated with various concentrations of cryptolepine (0, 2.5, 5.0 and 7.5 µM) for 24 h, then double staining was performed using phospho-p53- and cytochrome c specific primary antibodies following the immunohistochemistry protocol as detailed under Materials and Methods. Green color reflects the release of cytochrome c, red color shows the expression of P-p53 and DAPI shows blue. Representative photomicrographs are shown. Bar size = 5 µm; (**B**) SCC-13 or A431 cells were treated with different doses of cryptolepine (0, 2.5, 5.0 and 7.5 µM) for 24 h. Cells were incubated with rhodamine-123 for 30 min and then harvested for the analysis of mitochondrial membrane potential using Accuri Q6 flow cytometer. M1 compartment indicates percent of cells with intact mitochondrial membrane potential while M2 compartment indicates percent cells with loss of mitochondrial membrane potential.

In the event of DNA damage, activated p53 activates transcription of pro-apoptotic protein Bax and thus disrupt the balance of Bax/Bcl-2 protein ratio in cells and that results in release of cytochrome c from mitochondria leading to apoptosis [32,33,34]. In the present study, it can be clearly seen that cryptolepine-treated SCC-13 and A431 cells enhances the release of cytochrome c from the mitochondria, as indicated by the increased intensity of green color in immunohistochemical analysis (Figure 5A). Further, when cryptolepine treated cells (SCC-13 and A431) were evaluated for mitochondrial membrane potential using flow cytometry, an increased percentage of cell population with lost mitochondrial membrane potential (compartment M2) was observed compared to non-treated control cells, as shown in Figure 5B. The range of cell population having loss of mitochondrial membrane potential in SCC-13 cells was 3.9% to 42.6% compared to 1.0% in non-treated control cells, while in A431 cells it was 22.0% to 50.4% compared to 1.3% in non-treated control cells. These changes are important and decide the fate of cancer cells.

### 2.7. Cryptolepine Inhibits Cell Viability and Induces Apoptotic Cell Death in NMSC Cells

**Figure 6:**
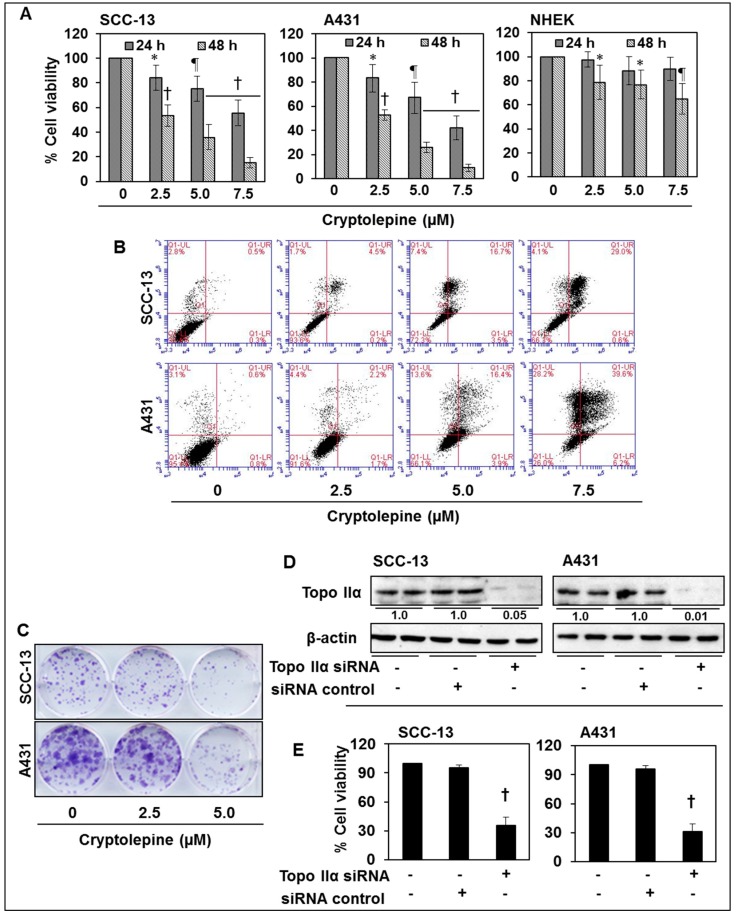
Treatment of cryptolepine inhibits cell viability, induces apoptosis and reduced colony formation capacity of NMSC cells. NMSC cells (SCC-13 and A431) and NHEK were treated with different concentrations of cryptolepine (0, 2.5, 5.0 and 7.5 µM) for 24 and 48 h. (**A**) Cell viability was determined using MTT assay. Experiment was performed in six individual wells of 96 wells plate and cell viability was compared with the control, *n* = 6. Statistical significance versus control, * *p* < 0.05; ^¶^ *p* < 0.01; ^†^ *p* < 0.001; (**B**) Cells were treated with various concentrations of cryptolepine (0, 2.5, 5.0 and 7.5 µM) for 24 h. Thereafter, cells were harvested, and incubated with Alexa488 reagents and PI for 30 min, percent apoptotic cell population was analyzed using Accuri Q6 flow cytometer, as described in Materials and Methods; (**C**) After treatment with cryptolepine (0, 2.5 and 5.0 µM) for 24 h, 500 NMSC cells were allowed to grow in 6-well plates in duplicate for 2 weeks at 37 °C in an incubator. After two weeks, colonies were identified using 0.5% crystal violet. Cell colonies were scanned for photographs, and are seen in blue; (**D**) Western blot analysis indicates that the levels of Topo IIα was markedly decreased in the NMSC cells after knocked-down of Topo IIα using siRNA kit; (**E**) Cell viability in SCC-13 and A431 cell lines was significantly decreased (*p* < 0.001) after knock-down of Topo IIα using siRNA kit compared to the cells treated with control siRNA.

As treatment of SCC-13 and A431 cells with cryptolepine resulted in inhibition of topoisomerase activity and stimulates DNA damage, it is expected that cryptolepine treatment will inhibit the cell viability/growth of these NMSC cells. Therefore, the effect of cryptolepine on cell viability of SCC-13, A431 and NHEK cells was determined using MTT assay. For this purpose, SCC-13, A431, and NHEK cells were treated with various concentrations of cryptolepine (0, 2.5, 5.0 and 7.5 µM) for 24 and 48 h. When compared with control treated cells, treatment of SCC-13 cells with cryptolepine resulted in a significant reduction (*p* < 0.05 to *p* < 0.001) in cell viability, and it ranged from 17% to 45% after 24 h, 47% to 85% after 48 h of treatment. More or less similar effects of cryptolepine were obtained on treatment of A431 cells (Figure 6A). In contrast, the sensitivity of the NHEK cells to the cytotoxic effects of cryptolepine was much lower than NMSC cells, with cryptolepine only having a significant inhibitory effect (*p* < 0.05 to *p* < 0.01) on the viability of the NHEK cells after 48 h of treatment. Moreover, the cryptolepine-induced inhibition of cell viability in NHEK cells at this dose and time point was significantly less (*p* < 0.01 to *p* < 0.005) than the effects of the same dose of cryptolepine on NMSC cells at the same time point (Figure 6A). Thus, results of cell viability assay suggested that cryptolepine is highly selective in inhibiting cell viability of skin cancer cells vs. normal cells. To further determine whether the cryptolepine induced loss of cell viability and DNA damage in the NMSC cells is associated with the induction of apoptosis, SCC-13 and A431 cells were treated with cryptolepine for 24 h and the percentage of apoptotic cells was determined using the Annexin V-conjugated Alexafluor488 (Alexa488) Apoptotic Detection Kit as described previously [35].

Cryptolepine treatment of SCC-13 cells for 24 h resulted in a significant dose-dependent enhancement in the percentage of apoptotic cells particularly at the late stage of apoptosis (Figure 6B, upper right panel). 0 µM (vehicle control, 0.5%), 2.5 µM (4.5%), 5.0 µM (16.7%) and 7.5 µM (29.0%). Similar results were obtained on cryptolepine treatment of A431 cells for 24 h (Figure 6B, lower panel).

Cryptolepine treatment of NHEK cells for 24 h did not result in significant enhancement of apoptosis of NHEK cells (data not shown). These data suggest that at least under the experimental conditions used in this study, cryptolepine is significantly less toxic to normal skin cells. Further, the cytotoxic effect of cryptolepine was also assessed in skin cancer cells using colony formation assay. As shown in Figure 6C, treatment of cells with cryptolepine reduced the colony formation abilities of SCC-13 and A431 cells demonstrating that cryptolepine is also effective in inhibition of long-term cell proliferation ability of non-melanoma skin cancer cells.

### 2.8. siRNA Knock-Down of Topo IIα in NMSC Cells Results in Inhibition of Cell Viability 

Further to verify the role of Topo IIα in NMSC cell growth, the level of Topo IIα was knocked down in the NMSC cells using siRNA kit, and cells were subjected to the analysis of cell growth/viability using MTT assay. We also checked the level of Topo IIα after its knock-down. Western blot analysis revealed that the levels of Topo IIα in both cell lines were decreased substantially after its knock down (Figure 6D). As shown in Figure 6E, the cell viability was also significantly decreased (*p* < 0.001) in both SCC-13 and A431 cell lines after the knock-down of Topo IIα, as analyzed by MTT assay.

## 3. Discussion

Topoisomerases are known to play a crucial role during DNA replication and cell proliferation, and their functions become irregular in cancer cells. Because of this reason, inhibition of topoisomerases activity is a central mechanism of action of various anticancer drugs, such as camptothecin, etoposide and doxorubicin, used in cancer chemotherapy [20,23]. These drugs inhibit topoisomerase functions and induce DNA stand breaks that lead to DNA damage, cell cycle arrest and induction of apoptosis [18,21,22]. In the present study, we have evaluated the chemotherapeutic effect of cryptolepine, a plant alkaloid, on topoisomerase function and DNA damage capacity using NMSC cells (SCC-13 and A431) as an in vitro model. Our study reveals that Topo I and Topo II expressions and their activities were higher in SCC-13 and A431 skin cancer cells compared to NHEK. However, treatment of cryptolepine decreases expression and activity of Topo I and Topo II in both SCC-13 and A431 cells. Since Topo IIα activity and functions are important for cellular functions and have been widely studied for anticancer activities [20,21], the effect of cryptolepine was determined for Topo IIα in NMSC. Induction of DNA damage is the central mechanism of topoisomerase inhibitors [18,23]. Results from comet assay reveals that cryptolepine treatment induces significant DNA damage in SCC-13 and A431 cells as is reflected from their tail lengths. Inhibition of topoisomerase activity and induction of DNA damage stimulates DNA repair enzymes. DNA-PK is a protein kinase involved in strand break repair and gets activated in the event of topoisomerase inhibition or chemical induced DNA damage [26]. Double staining of Topo IIα and DNA-PK in cryptolepine treated or untreated NMSC cells revealed that it inhibits topoisomerase expression while enhances DNA repair enzyme DNA-PK. DNA damage response pathway includes damage sensors, signal transducers, and effectors [36,37]. DNA damage triggers activation of DNA damage response elements, such as ATM and ATR. Activation of ATR is usually associated with damage to single-strand DNA or stalled DNA replication forks while activation of ATM is associated with initiation of signaling pathways in response to double strand breaks [37,38]. We have found that treatment of NMSC cells with cryptolepine induces phosphorylation of both ATM and ATR proteins in SCC-13 and A431 cells. During inhibition of topoisomerase activity, activated ATM and ATR directly or through sequential steps phosphorylate downstream proteins BRCA1, γH2AX, Chk1 and Chk2 and subsequently affect downstream factors involved in cell cycle progression and cell survival [18,21,22]. Phosphorylated γH2AX and BRCA1 are involved in DNA repair and activation of other repair factors, whereas, phosphorylated Chk1 and Chk2 activate factors involved in cell cycle arrest and apoptosis [30]. As a consequence of cryptolepine induced DNA damage in SCC-13 and A431 cells, BRCA1, γH2AX, Chk1 and Chk2 were greatly phosphorylated. Phosphorylation of BRCA1, γH2AX, Chk1 and Chk2 observed in cryptolepine treated cells is also supported by the evidences that have demonstrated that clinically used cancer chemotherapeutic agents which inhibit topoisomerase functions also activate these signaling cascade [20,23].

The tumor suppressor protein p53 is a crucial component of cellular machinery that regulates various signaling pathways including oncogenic processes, cell cycle, apoptosis and DNA damage responses under different conditions. Under normal conditions, in unstressed cells, the expression and function of p53 are tightly regulated and maintained in low levels with short half-life [28,39]. However, under stressed conditions, such as induction of DNA damage, nucleotide depletion, or hypoxia, levels of p53 protein increases significantly. The mechanism of enhanced p53 levels after DNA damage is believed to be post-translational modifications such as phosphorylation and acetylation [28,40,41]. In response to topoisomerase inhibition or chemically induced DNA damage, activated ATM or Chk2 directly activates p53 by phosphorylation or inhibits its interactions with negative regulator mdm2. Mdm2 protein attenuates p53 activity either through auto-regulatory loop by interacting with amino terminus of p53 or by activating degradation process. CDK inhibitory proteins p21 and p16 are major downstream proteins transcriptionally activated by p53. Enhanced expression of p21 and p16 proteins inhibits cell cycle progression and induces apoptosis [36,42]. Results from our experiments clearly demonstrates that cryptolepine induced topoisomerase inhibition and induction of DNA damage in SCC-13 and A431 cells resulted in activation and accumulation of p53 protein via enhanced phosphorylation and acetylation. Cryptolepine treatment also down regulates the level of mdm2 protein in these cells. Moreover, expression of p16 and p21 was also enhanced due to activation of p53 in these cells after cryptolepine induced DNA damage. Furthermore, activated p53 and p16 and p21 proteins interact with cyclins, Cdc and CDKs involved in regulation of cell cycle progression. Different phases of cell cycle are tightly regulated by specific interactions of CDKs, Cdc and cyclins [43,44,45]. Activated p16 specifically inhibits binding of CDK2/cyclin A resulting in G1/S transition arrest whereas p21 protein, a universal inhibitor of CDKs, inhibits interaction with cyclin E. The induced p21 protein binds to both the CDK2/cyclin A and CDK2/cyclin E [42,46,47]. Western blot analysis demonstrated activation of p16 and p21 after treatment with cryptolepine and that is associated with the inhibited expression of Cdc25a/b, cyclin A/E and CDK2 compared to non-treated control cells suggesting the inhibition of cell cycle progression. Analysis of cell cycle phase distribution by flow cytometry confirmed cryptolepine induced cell cycle arrest at S-phase in NMSC cells. Activated p53 is also capable to induce Bax expression and thus imbalance Bax/Bcl-2 ratio in cells, which in turn initiates mitochondrial membrane potential disruption-mediated apoptosis in cells through release of cytochrome c from mitochondria [33,48]. We also found that cryptolepine induced DNA damage and phosphorylation of p53 leads to cytochrome c release from mitochondria. The results were further confirmed by flow cytometry which shows that cryptolepine induced DNA damage was related with loss in mitochondrial membrane potential and subsequently release of cytochrome c. These cryptolepine-induced changes in NMSC cells resulted in significant decrease in cell viability and induction of apoptosis in SCC-13 and A431 cells. Cryptolepine was significantly less toxic to NHEK compared to NMSC cells demonstrating that cells expressing higher topoisomerase expression and activity are more susceptible to cryptolepine-induced cytotoxicity. Further, it was observed and verified that the knock down of Topo IIα in both cell lines results in significant reduction in cell viability, and thus shows the importance of Topo IIα in the growth and progression of NMSC cells. In addition, cryptolepine treatment inhibits long-term cell proliferation ability in NMSC cells as observed in colony formation assay.

**Figure 7:**
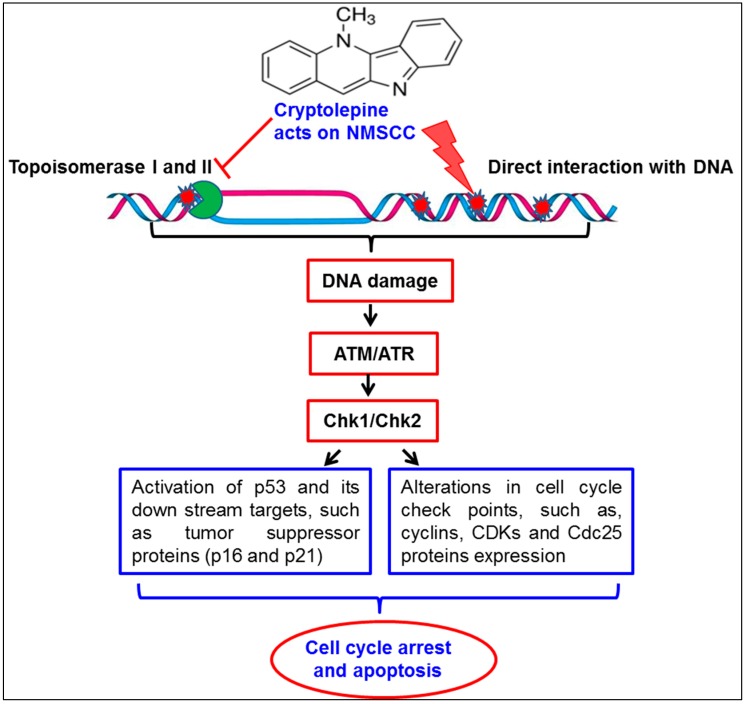
Schematic representation of cryptolepine-induced effects on non-melanoma skin cancer cells (NMSCC). Cryptolepine inhibits topoisomerase and causes DNA damage in skin cancer cells. Cryptolepine induced DNA damage response leads to activation of ATM/ATR and checkpoint kinases (Chk1/Chk2) resulting in activation of p53 signaling and modulation of cell cycle regulatory proteins. Together, these changes induced by cryptolepine results in cell cycle arrest and ultimately induction of apoptotic cell death of NMSCC.

In summary, cryptolepine, a pharmacologically active plant alkaloid, possesses strong anticancer potential against non-melanoma skin cancer cells in an in vitro model. The anticancer activity of cryptolepine is mediated through topoisomerase inhibition and subsequently induction of DNA damage. Cryptolepine induced DNA damage response signaling through activation of p53 pathway leading to cell cycle arrest and induction of apoptosis in skin cancer cells, as depicted and summarized in Figure 7. Since topoisomerase inhibition and induction of cancer cell death by activating DNA damage is the most common mechanism of action of many current chemotherapeutic anticancer drugs, cryptolepine has a promise to be tested further at least in preclinical animal model for the assessment of its anticancer activities.

## 4. Materials and Methods

### 4.1. Cryptolepine, Chemicals, Reagents and Antibodies

Cryptolepine with ≥98% purity was purchased from Sigma-Aldrich (St. Louis, MO, USA). Topoisomerase I and II assay kits were obtained from TopoGEN, Inc. (Buena Vista, CO, USA). Primary antibodies specific for topoisomerase I and IIα were purchased from Abcam (Cambridge, MA, USA). Phospho forms of DNA damage specific primary antibodies of ATM, ATR, BRCA1, Chk1, Chk2, γH2AX, p53 and, acetylated-p53 were purchased from Cell Signaling Technology (Beverly, MA, USA). Antibodies for total p53, mdm2, DNA-PK, cytochrome c, Cdc25a, Cdc25b, p16, p21, CDK2, cyclin A, cyclin D1, cyclin E, vinculin, β-actin, HRP-conjugated anti-mouse, anti-rabbit, and anti-goat were purchased from Santa Cruz Biotechnology, Inc. (Santa Cruz, CA, USA). Apoptotic cells were analyzed using Annexin-V Alexa Fluor^®^ 488 (Alexafluor488) kit from Molecular Probes (Eugene, OR, USA).

### 4.2. Cells and Cell Culture Conditions

The human squamous cell carcinoma cell lines (SCC-13 and A431), immortalized human skin keratinocyte cell line (HaCaT) and primary normal human epidermal keratinocytes (NHEK) were purchased from the American Type Culture Collection (ATCC, Manassas, VA, USA). SCC-13, A431 and HaCaT cells were cultured as monolayers in Dulbecco’s modified Eagle’s medium supplemented with 10% heat inactivated fetal bovine serum, 100 μg/mL penicillin–streptomycin, and maintained in a humidified atmosphere of 95% air and 5% CO_2_ at 37 °C. NHEK cells were grown in dermal cell basal media (ATCC^®^ PCS-200-030) supplemented with keratinocyte growth factors (ATCC^®^ PCS-200-040) and maintained under standard cell culture conditions. Cell lines were authenticated by the vendor and each cell line was passaged up to 3–4 times for experimental purposes. The pathological information of cancer cell lines are as follows: A431 cells are epidermoid carcinoma cells with epithelial morphology. These cells were isolated from the skin tumor tissue of female patient. SCC-13 cells were isolated from the skin squamous cell carcinoma from a female patient. Cryptolepine was dissolved in DMSO with final concentration of DMSO in media was not more than 0.1% (*v*/*v*). Equal volume of DMSO was added in control group of cells.

### 4.3. Preparation of Topoisomerase (I & II) Extract from Cells

Cells from treated and untreated groups were harvested and collected into 5 mL medium in 15 mL tubes. Suspensions were centrifuged at 800× *g* for 3 min at 4 °C. Cell pellets were resuspended in 3 mL of ice cold TEMP buffer (10 mM Tris-HCl, pH 7.5, 1 mM EDTA, 4 mM MgCl_2_ and 0.5 mM PMSF). Suspensions were centrifuged at 800× *g* for 3 min at 4 °C and cell pellets were resuspended in 3 mL of ice cold TEMP buffer and left on ice for 10 min. After incubation, suspensions were homogenized by 8–10 strokes in Dounce tight fitting homogenizer. Homogenates were centrifuged at 1500× *g* for 10 min at 4 °C to pellet the nuclei. Nuclear pellets were resuspended in 1 mL of cold TEMP buffer in a microcentrifuge tube. Tubes were centrifuged at 1500× *g* for 2 min at 4 °C, pellets collected and resuspended in a small volume (~3 pellet volume) of TEP buffer (same as TEMP buffer but lacking MgCl_2_. An equal volume of 1M NaCl was added, vortexed and left on ice for 60 min. After incubation, tubes were centrifuged at 15,000× *g* for 15 min at 4 °C. The supernatants were collected as topoisomerase (I & II) extracts. Topoisomerase activity assays were performed on same day or extracts were aliquoted and stored at −80 °C. Protein concentration was determined using a Bio-Rad protein assay kit (Bio-Rad Laboratories, Inc., Hercules, CA, USA).

### 4.4. Topoisomerase I Enzyme Activity Assay

To determine the topoisomerase I enzyme activity, cell extracts containing 10 µg protein from treated or untreated groups were mixed with 2 µL of 10x topoisomerase I assay buffer (100 mM Tris-HCl pH 7.9, 10 mM EDTA, 1.5 M NaCl, 1% bovine serum albumin, 1 mM spermidine, 50% glycerol) and 1 µL (0.25 µg/ µL), and supercoiled DNA (25 µg in 100 µL TE buffer; 10 mM Tris-HCl pH 7.5, 1 mM EDTA) as a substrate. Reaction volumes were made up to 20 µL using distilled water. Reaction mixtures were incubated for 30 min at 37 °C. Reaction was stopped by adding 5 µL of 2% SDS. Proteinase K (0.5 mg/mL) 5 µL was added and incubated for 15 min at 37 °C. Reaction mixture with 10x loading dye (0.25% bromophenol blue, 50% glycerol) was loaded to 1% agarose gel in TAE buffer. Gel was run at 1–2.5 V/cm for 2 h. Gel was stained with 0.5 µg/mL ethidium bromide and destained in distilled water for 15 min at room temperature and photographed using UV transilluminator from Bio-Rad. Comparative reactivity of the enzyme among different groups is represented by the band intensity. 

### 4.5. Topoisomerase II Enzyme Activity Assay

To determine the topoisomerase II enzyme activity, cell extracts equivalent to 10 µg protein each from treated or untreated groups were mixed with 4 µL of 5x complete topoisomerase II assay buffer prepared freshly by mixing equal volume of Buffer A (0.5 M Tris-HCl pH 8, 100 mM MgCl_2_, 5 mM dithiothreitol, 300 µg bovine serum albumin/mL) and Buffer B (200 mM ATP in sterile distilled water). One µL (0.25 µg/ µL) supercoiled pHOT1 DNA (150 ng to final volume) was added as a substrate. Reaction volumes were made up to 20 µL using distilled water. Reaction mixtures were incubated for 30 min at 37 °C. Reaction was stopped by adding 5 µL of 2% SDS. Proteinase K (0.5 mg/mL) 5 µL was added and incubated for 15 min at 37 °C. Reaction mixture with 10x loading dye (0.25% bromophenol blue, 50% glycerol) was loaded to 1% agarose gel in TAE buffer. Gel was run at 1–2.5 V/cm for 2 h. Gel was stained with 0.5 µg/mL ethidium bromide and destained in distilled water and photographed using UV transilluminator from Bio-Rad. Comparative reactivity of the enzyme among different treatment groups is represented by the band intensity.

### 4.6. Knockdown of Topo IIα Expression in NMSC Cells Using siRNA

Topo IIα expression in SCC-13 and A431 cells was knocked-down by transfection with human-specific Topo IIα siRNA Kit (Santa Cruz Biotechnology). Transfection was performed according to the manufacturer’s instructions. Briefly, 2 × 10^5^ cells were seeded in each well of 6-well plate and allowed to grow to 60%–70% confluency. The Topo IIα siRNA mixed with transfection reagents was overlaid on the cells and incubated at 37 °C. After 8 h, cells were incubated with 2x growth medium for about 16–18 h. At 24 h post transfection medium was replaced with fresh medium and further incubated for additional 48 h. Thereafter, cells were harvested and cell lysates prepared for western blot analysis to check the levels of Topo IIα. siRNA transfected cells were also analyzed for cell viability using MTT assay.

### 4.7. Analysis of DNA Damage by Comet Assay

Cryptolepine-induced DNA damage in SCC-13 and A431 cells was determined using comet assay, as described in detail previously [49,50]. DNA damage was detected and images were obtained under an Olympus microscope (Model BX41TF, Olympus Corporation, Tokyo, Japan) equipped with a Q-Color 5 camera with CellSens software. In each treatment group, DNA tail length was determined using opencomet software and expressed as a mean ± SD.

### 4.8. Preparation of Cell Lysates and Western Blot Analysis

After 24 h treatment with or without cryptolepine, cells were harvested and cell lysates were prepared as described previously [51,52]. Briefly, equal amount of proteins were electrophoretically resolved on tris-glycine gels and transferred onto a nitrocellulose membrane. Non-specific sites were blocked by incubating the membrane with blocking buffer for 1 h. The membrane was incubated with specific primary antibodies overnight at 4 °C followed by 2 h incubation with HRP-conjugated secondary antibodies. The equal loading of proteins in each sample was verified by reprobing the stripped membrane with housekeeping genes anti-β-actin or anti-vinculin antibodies. Most of the data on western blot analysis are presented from two separate experiments. Same β-actin bands may be presented more than once if same data are generated from the same membrane. The relative density of each band in a blot was measured using the ImageJ software (National Institutes of Health, Bethesda, MD, USA). The numerical value of band density is shown under blot, and the band density of control group (non-treatment group) was arbitrarily selected as ‘1’ and comparison was then made with densitometry values of other treatment groups. Further, as the immunoblot data are presented separately from two independent experiments under each treatment group, we are showing the mean value of two bands from two different experiments under each treatment group. 

### 4.9. Immunofluorescence Staining

Approximately 5 × 10^4^ SCC-13 or A431 cells/well were seeded in four well chambered slides. Next day, cells were treated with cryptolepine (0, 2.5, 5.0 and 7.5 µM) for 24 h. After incubation, cells were washed twice with chilled PBS, fixed with 4% paraformaldehyde and permeabilized with 0.5% Triton-X 100 in PBS for 3 min. Nonspecific binding was blocked by incubating cells with 3.0% BSA in PBS for 30 min. Cells were incubated with specific primary antibodies over night at 4 °C. Cells were washed with PBS and incubated further for 1 h with fluorochrome conjugated secondary antibodies. After washing with PBS, slides were mounted with Vectashield^®^ mounting medium (Vector Laboratories, Inc. Burlingame, CA, USA) containing DAPI, analyzed and imaged using an Olympus microscope.

### 4.10. Cell Cycle Analysis

NMSC cells (SCC-13 or A431) were treated with different concentrations of cryptolepine (0, 2.5, 5.0 and 7.5 µM) for 24 h. The cells were then harvested, and processed for cell cycle analysis, as described previously [53]. Briefly, the cells were fixed in chilled 70% methanol overnight at 4 °C. After centrifugation, the cells were washed with chilled PBS and then incubated with RNase A (20 µg/mL) for 30 min. The cells were then incubated with propidium iodide (50 µg/mL) for at least three h in dark at 4 °C. The cell cycle phase distribution of the cells was then determined using an Accuri Q6 flow cytometer (BD Biosciences, San Jose, CA, USA).

### 4.11. Mitochondrial Membrane Potential Analysis

Retention of rhodamine 123 dye by mitochondria was performed for determining the change in mitochondrial membrane potential, as described previously [54]. Approximately 2 × 10^5^ SCC-13 or A431 cells were treated with different doses of cryptolepine (0, 2.5, 5.0 and 7.5 µM) for 24 h. Cells were incubated with rhodamine 123 for 30 min and then harvested, washed with PBS and resuspended in PBS for analysis of mitochondrial membrane potential using an Accuri Q6 flow cytometer.

### 4.12. MTT Assay For Cell Viability

The MTT assay was employed to determine the effect of cryptolepine on cell viability, as described previously [55]. Briefly, approximately 1 × 10^4^ cells/well were plated in 96-well culture plates. The cells in each treatment group were plated at least in 8 replicates. Next day, cells were treated with different concentrations of cryptolepine (0, 2.5, 5.0 and 7.5 µM) for 24 and 48 h. After incubation with indicated time periods, media was replaced with 50 µL fresh medium containing 5 mg/mL MTT and incubated for 2 h in incubator. The resulting formazan crystals were dissolved in 200 µL DMSO. Absorbance was recorded at 540 nm with a reference at 650 nm serving as the blank. The effect of cryptolepine on cell viability was presented in terms of percent of vehicle-treated control cells. The viability of control cells were arbitrarily considered as 100%.

### 4.13. Apoptotic Cell Death Analysis

Quantitative analysis of cryptolepine-induced apoptosis in SCC-13 and A431 cells was determined by flow cytometer using Annexin V-conjugated Alexa fluor488 (Alexa488) Apoptosis Detection Kit following the manufacturer’s protocol, and as described previously by us [35,55]. Briefly, 1 × 10^6^ cells were treated with cryptolepine (0, 2.5, 5.0 and 7.5 µM) for 24 h. After incubation, cells were harvested, washed with PBS and incubated with Alexa488 and propidium iodide. The apoptotic cells were analyzed by an Accuri C6 flow cytometer.

### 4.14. Cell Colony Formation Assay

The effect of cryptolepine on long-term cell proliferation/viability (clonogenic potential) was determined by colony formation assay, as described previously [35]. Briefly, 500 cells from each of cryptolepine treated and un-treated group (0, 2.5, 5.0 µM for 24 h) were suspended in 3 mL complete growth medium media, plated individually in separate wells of 6-well plate. Cells were allowed to grow for total 14 days, while media was replaced on 7th day. On 14th day, colonies were washed with chilled PBS, and fixed in chilled methanol for 10 min. Colonies were stained with 0.5% crystal violet (made in 25% methanol) for 10 min and washed three times with water to remove excess of dye. Colonies were air dried, and plates were scanned for photographs. 

### 4.15. Statistical Analysis

The statistical significance of the difference between the values of control and treatment groups was determined using student-t test and one-way analysis of variance (ANOVA) using GraphPad Prism version 4.00 for Windows (GraphPad Software, San Diego, CA, USA; www.graphpad.com). In each case, *p* < 0.05 was considered as statistically significant.
